# Enhanced Acetone Sensing Characteristics of ZnO/Graphene Composites

**DOI:** 10.3390/s16111876

**Published:** 2016-11-09

**Authors:** Hao Zhang, Yuan Cen, Yu Du, Shuangchen Ruan

**Affiliations:** 1 Shenzhen Key Laboratory of Laser Engineering, College of Optoelectronic Engineering, Shenzhen University, Shenzhen 518060, China; haozhang@szu.edu.cn; 2 Key Laboratory of Optoelectronic Devices and Systems of Ministry of Education and Guangdong Province, College of Optoelectronic Engineering, Shenzhen University, Shenzhen 518060, China; 3 Shenzhen Key Laboratory of Sensor Technology, College of Physics Science and Technology, Shenzhen University, Shenzhen 518060, China; cenyuan900927@163.com

**Keywords:** graphene, acetone sensing, ZnO

## Abstract

ZnO/graphene (ZnO-G) hybrid composites are prepared via hydrothermal synthesis with graphite, N-methyl-pyrrolidone (NMP), and Zn(NO_3_)_2_·6H_2_O as the precursors. The characterizations, including X-ray diffraction (XRD), thermogravimetric analyses (TGA), Raman spectroscopy, and transmission electron microscopy (TEM) indicate the formation of ZnO-G. Gas sensors were fabricated with ZnO-G composites and ZnO as sensing material, indicating that the response of the ZnO towards acetone was significantly enhanced by graphene doping. It was found that the ZnO-G sensor exhibits remarkably enhanced response of 13.3 at the optimal operating temperature of 280 °C to 100 ppm acetone, an improvement from 7.7 with pure ZnO.

## 1. Introduction

Chemical sensors play an important role in the areas of emissions control, environmental protection, public safety, and human health [1]. It is well known that high sensitivity, fast response and recovery, and selective detection are required for real-time monitoring of harmful gases and preventing possible disasters due to toxic gas [2,3,4]. Among them, the detection of acetone vapor is very important in daily life. Medical investigations have shown that the acetone concentration in exhaled breath from a healthy human body is lower than 0.8 ppm, while that for a diabetic patient is higher than 1.8 ppm [5,6,7]. As an important chemical material, acetone vapor should be monitored, and kinds of metal oxides have been applied. Zinc Oxide—as an n-type semiconductor material—has been widely investigated as a field-effect transistor [7], optical device [8], dye-sensitized solar cell [9], and solid-state gas sensor [10,11]. Recently, ZnO-based sensors have been investigated for the detection of acetone vapor at various concentration levels [12,13,14,15].

Graphene, known as “the thinnest material in our universe”, with only one-atom thickness, has attracted huge attention since its discovery. Because of its unique features of high surface area, light weight, high electron mobility, and mechanical strength, graphene can make a highly promising platform for gas detection. In order to improve the sensing performances of graphene-based sensors, various sensitive materials have been selected to modify the graphene conductive network, and they play important roles in improving the sensitivity and selectivity of resultant gas sensors [16,17,18,19]. To date, the most popular method of preparing monolayer or multilayer graphene is the micromechanical cleavage or chemical exfoliation of highly oriented pyrolytic graphite or graphite oxide [20,21,22,23,24,25]. Recently, exfoliated graphene was prepared directly from graphite with solvothermal method, offering another method for graphene preparation [26]. Meanwhile, to the best of our knowledge, the enhanced acetone sensing performance of the ZnO/graphene (ZnO-G) composites prepared by this method has yet to be reported.

Hence, we present a simple and economically attractive route for the synthesis of the ZnO-G composites via a facile hydrothermal method on a large scale. Simultaneously, to improve the acetone sensing performance, the graphene was exfoliated directly from graphite by solvothermal treatment. Improved gas sensitivity and selectivity of ZnO-G towards acetone was achieved. The effects of graphene doping on the response and response-recovery time at different operating temperatures and gas concentration towards acetone were also investigated.

## 2. Materials and Methods

### 2.1. Chemicals

All chemicals were of analytical grade and were used as received without further purification. Graphite and N-methyl-pyrrolidone (NMP) and Zn(NO_3_)_2_·6H_2_O, were supplied by Beijing Chemical Corp, Ltd. (Beijing, China). The water used throughout all experiments was purified through a Millipore system.

### 2.2. Exfoliation of Graphene

The exfoliation of Graphene has been proposed [27]. In a typical synthesis, 0.5 g of graphite was dispersed in 35 mL of NMP solvent, which was then sealed into a 40 mL Teflon-lined autoclave and solvothermally treated at 200 °C for 3 days. The autoclave was cooled naturally, and the as-obtained sample was sonicated for 1 h in a sonication bath. After the removal of macroscopic aggregates and a thick layer of graphene by centrifugation (5–7 krpm for 7–10 min), a dark suspension was obtained. After a few minutes, a graphene film was formed at the interface.

### 2.3. Preparation of ZnO-G Composites

ZnO-G composite was prepared by in situ production of ZnO nanoparticles on the surface of graphene. In a typical synthesis, 2.19 g Zn(NO_3_)_2_·6H_2_O and 6 mL of graphene of NMP resolution (1 mg/mL) was introduced into 20 mL H_2_O, which was sonicated for 40 min. The aqueous dispersion was transferred into a 40 mL Teflon-lined stainless-steel autoclave and heated at 180 °C for 12 h. The black product was harvested by centrifugation and washed with water and ethanol several times, and dried at 60 °C for several hours. For comparison, the pure ZnO was prepared by a similar method without the addition of graphene.

### 2.4. Characterizations

Powder X-ray diffraction (XRD) data were recorded on a Rigaku D/Max-2550 diffractometer with Cu-Kα radiation (λ = 0.15418 nm). The transmission electron microscopic (TEM) images were taken with a JEOL JEM-3010 TEM microscope with an accelerating voltage of 200 kV. The sample for TEM characterization was prepared by placing a drop of colloidal solution on a carbon-coated copper grid and dried at room temperature. Thermogravimetric analyses (TGA) analysis was measured on ATGAQ50 instrument from 25 °C to 800 °C. Raman spectra were obtained on Horiba-JY T64000 Raman spectromer with 514.5 nm wavelength incident laser light.

### 2.5. Fabrication and Gas Sensing Measurements

**Figure 1:**
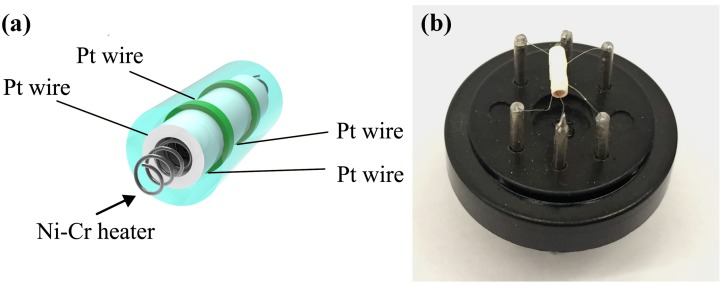
(**a**) A schematic image and (**b**) photograph of the ZnO/graphene (ZnO-G) sensor.

The product was mixed with deionized water at a weight ratio of 4:1 to form a paste. The sensor was fabricated by coating a ceramic tube with the paste to form a 1.5 mm sensing film. A pair of gold electrodes was installed at each end of the ceramic tube before it was coated with the paste. Each electrode was connected with two Pt wires. A Ni-Cr heating wire was inserted into the tube to form an indirect-heated gas sensor. Figure 1a,b show a schematic image of the as-fabricated sensor and a photograph of the sensor on the socket, respectively. The Pt wires between two electrodes were almost covered by the sensing material and could not contact target gas, and thus have no effect on the sensing property. The gas sensing properties of the sensor were measured by a CGS-8 series Intelligent Test Meter. The sensors were all heat-treated in 300 °C before gas sensing test to remove the solvent from the sensing material. The response of the sensor is defined as the ratio of sensor resistance in a target gas (R_g_) to that in dry air (R_a_) between 180 and 360 °C. The time taken by the sensor to achieve 90% of the total resistance change was defined as the response time in the case of adsorption or the recovery time in the case of desorption.

## 3. Results

### 3.1. Structural and Morphological Characteristics

**Figure 2:**
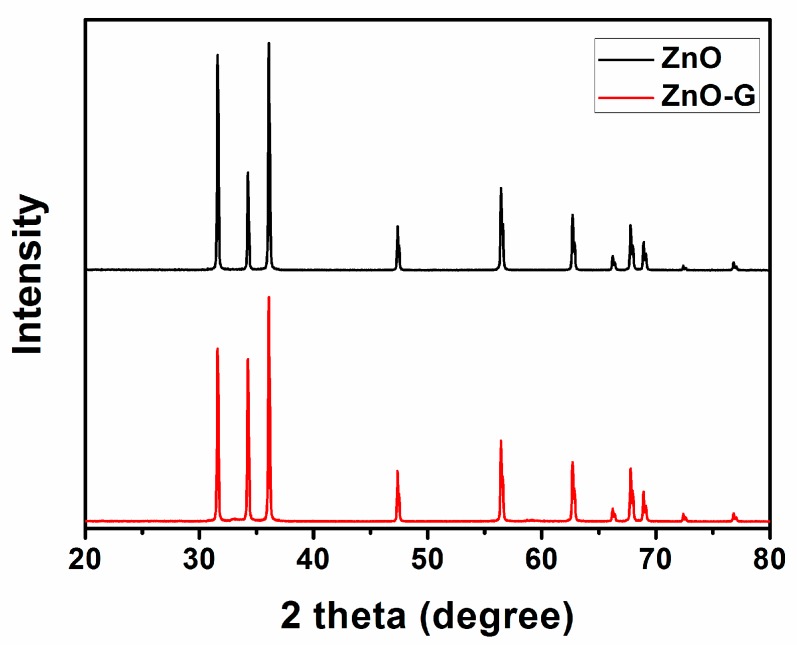
The powder X-ray diffraction (XRD) patterns of the products: ZnO (**black line**) and ZnO-G (**red line**).

The powder X-ray diffraction (XRD) pattern of the as-prepared product is shown in Figure 2. All diffraction peaks can be indexed to wurtzite-structured (hexagonal) ZnO (JCPDS No. 75-0576). No impurity phases were observed from the XRD pattern, which confirms the superb purity of the product. No graphene peak was observed; this may be due to the low content of graphene in the composite materials.

**Figure 3:**
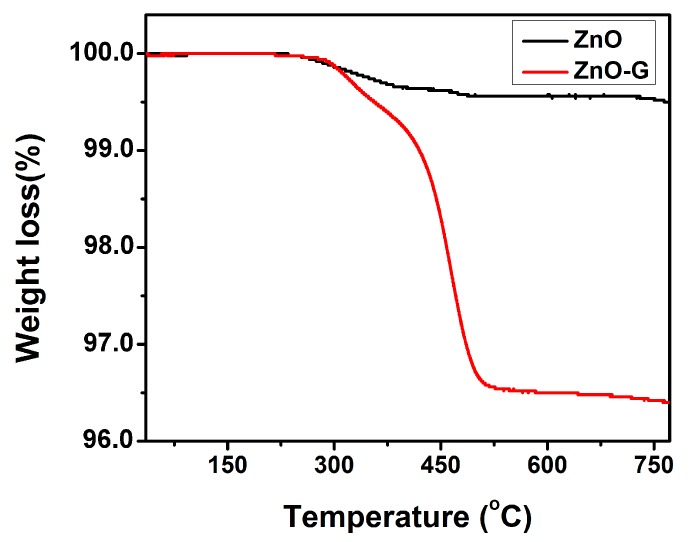
Thermogravimetric analysis (TGA) of ZnO and ZnO-G samples.

Figure 3 shows the TGA curves of ZnO and ZnO-G samples. The small weight loss observed in ZnO was 0.47%, attributed to desorption of solvent molecules physically adsorbed on the materials. Comparably, the weight loss of ZnO-G from room temperature to 325 °C is attributed to the removal of surface-bound water and NMP; the decomposition of the carbon framework started from 325 °C and continued up to 600 °C [28,29]. Based on the above results, the content of ZnO in ZnO-G hybrids is about 96.4%.

**Figure 4:**
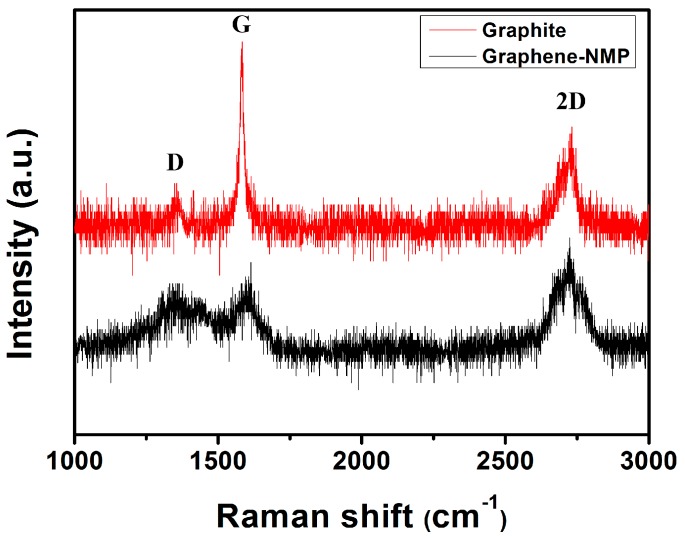
Raman spectra of graphite and graphene.

Figure 4 shows the Raman spectra of graphene and pristine graphite. The two strong peaks of the D peak (~1351 cm^−1^) and G peak (~1595 cm^−1^) were observed, corresponding to the diamondoid and graphitic graphene structures, respectively. The obtained D peak of graphene is higher than that of graphite, which indicates that the graphene that separated from the solvent may have more defects than graphite. This can be explained as below: although graphene was directly exfoliated from graphite, it will have some defects after exfoliation. However, the 2D lines (~2700 cm^−1^) of the two samples are nearly the same, which illustrates that the graphene that is collected has a majority of layered graphene [30].

**Figure 5:**
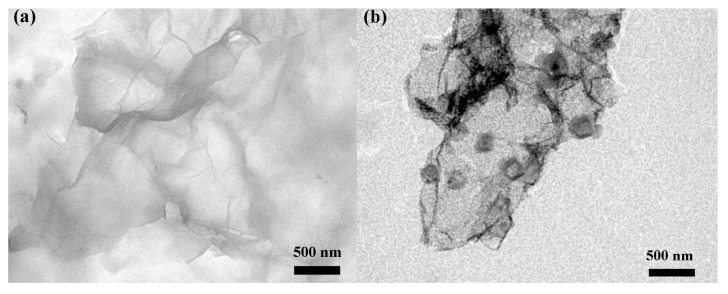
Transmission electron microscopy (TEM) images of (**a**) exfoliated graphene and (**b**) ZnO-G.

To observe the dispersions in NMP, TEM is employed by placing drops of the dispersions on microgrids. Figure 5a shows TEM images of representative graphene prepared by solvothermal treatment. Some flakes tend to crinkle or roll because of stress from the edges, and are similar to those prepared without solvothermal treatment. A TEM image of ZnO-G (Figure 5b) exhibits a typical morphology similar to graphene-based materials. Graphene with the size of about 3–4 µm is seen, and a few hexagonal ZnO nanoparticles with size 200–300 nm are decorated on the surface of graphene, indicating the formation of ZnO-G hybrids. This reveals that hydrothermal treatment of exfoliated graphene and Zn(NO_3_)_2_·6H_2_O solution is an effective method for the preparation of ZnO-G nanocomposite.

### 3.2. Acetone Sensing Properties

**Figure 6:**
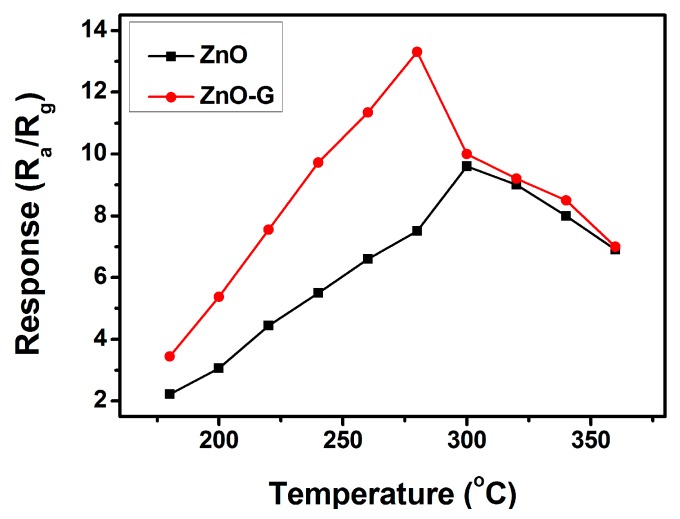
Response versus operating temperature of ZnO and ZnO-G sensors exposed to 100 ppm of acetone.

The responses of the sensors using pure ZnO and ZnO-G to 100 ppm acetone were measured at various temperatures in order to find the optimum operating temperature. As shown in Figure 6, the maximum response of the sensor based on pure ZnO was at 300 °C. Comparatively, the sensitivity of the ZnO-G sensor reached 13.3 at 280 °C. The response increases with increasing operating temperature up to 280 °C, since sufficient thermal energy is essential to overcome the activation energy barrier of chemisorption and surface reaction. Beyond 280 °C, the sensor sensitivity decreases, which may be due to the fact that the amount of adsorbed gas on the surface of the material has decreased, while the desorption process becomes dominant with increasing operating temperature, leading to a reduction in sensitivity. When the desorption rate of the gas becomes equal to that of adsorption, the maximum loading of chemisorbed ions is reached at the optimum temperature, accelerating the oxidation of acetone molecules and resulting in the highest sensitivity [31]. ZnO-G sensor exhibits relatively higher sensitivity and lower optimum sensing temperature than that of pure ZnO.

**Figure 7:**
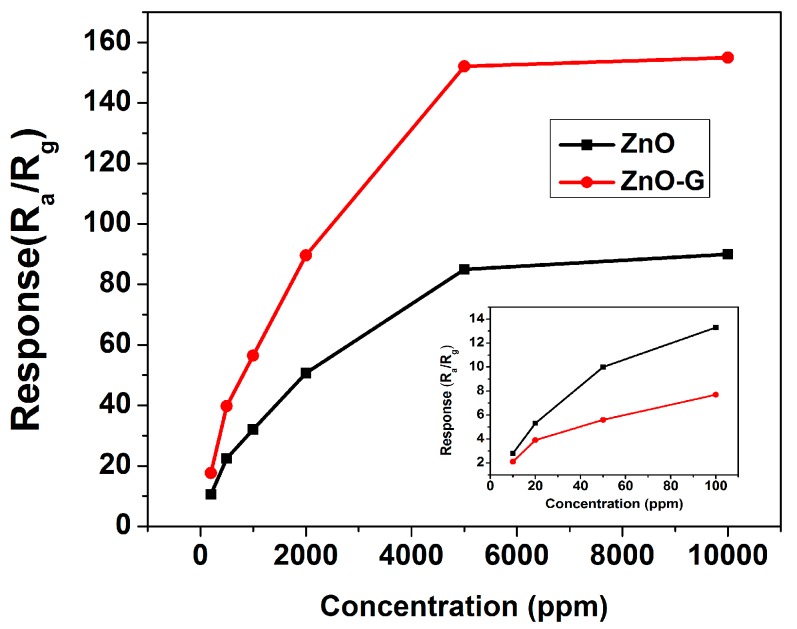
The responses of ZnO and ZnO-G sensors to different concentrations of acetone. The inset: the responses to acetone in the range of 10–100 ppm at 280 °C.

Figure 7 shows the response of the sensors based on ZnO and ZnO-G to acetone in the range of 10–10,000 ppm at 280 °C. The response of both sensors to acetone increases rapidly with the increasing of gas concentration below 5000 ppm, and increases slowly above 10,000 ppm. This indicates that its adsorption tends to saturation, and the upper concentration limit for acetone detection is 5000 ppm. Notably, the ZnO-G shows higher sensitivity to acetone in the entire range of detected concentration compared to ZnO, which indicates that the sensor is very suitable for the detection of acetone in a wide range of concentrations. This unsaturation phenomenon to relatively high acetone concentration (<5000 ppm) may result from the high surface area of ZnO-G, providing abundant surface active sites and absorbing a large amount of target gas molecules.

**Figure 8:**
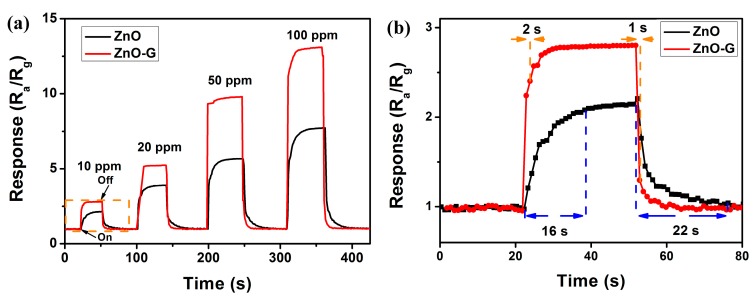
(**a**) Dynamic acetone sensing transients curve of the ZnO (**black line**) and ZnO-G (**red line**) sensor to 10–100 ppm acetone gases at 280 °C; (**b**) An enlarged image of the selected area of Figure 8a.

It is well known that the response and recovery characteristics are important for evaluating the performance of gas sensors. To investigate the response and recovery behaviors of ZnO and ZnO-G, the sensor was sequentially exposed to 10, 20, 50 and 100 ppm acetone at 280 °C. As seen in Figure 8, compared to ZnO, ZnO-G exhibits faster response and recovery behaviors to acetone. The response of ZnO-G is about 2.80, 5.20, 9.79 and 13.30, while that of ZnO is 1.40, 3.90, 5.65 and 7.72 to 10, 20, 50 and 100 ppm acetone, respectively (Figure 8a). To compare the response and recovery behaviors of ZnO and ZnO-G, an enlarged image of the gas sensing process to 10 ppm acetone is shown (Figure 8b). The response and recovery times of ZnO-G are about 1 and 2 s, and are 16 s and 22 s for ZnO, indicating the faster response and recovery behaviours of ZnO. The excellent response and recovery behavior can be explained by the high electron mobility of ZnO-G sensors. 

**Figure 9:**
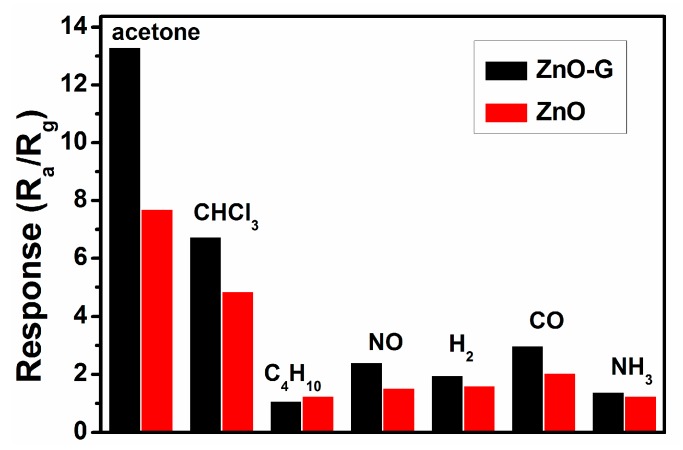
The response of the ZnO-G sensor to 100 ppm of different gases at 280 °C.

Figure 9 shows the response of ZnO and ZnO-G sensors at 280 °C to 100 ppm of various interference vapours. The responses of the ZnO sensor to CHCl_3_, C_4_H_10_, NO, H_2_, CO and NH_3_ are 4.86, 1.25, 1.52, 1.60, 2.05 and 1.26, respectively, and for ZnO-G are 6.75, 1.08, 2.40, 1.96, 2.98 and 1.39, indicating that graphene doping enhanced the selectivity of the ZnO-G sensor compared to the ZnO sensor.

**Figure 10:**
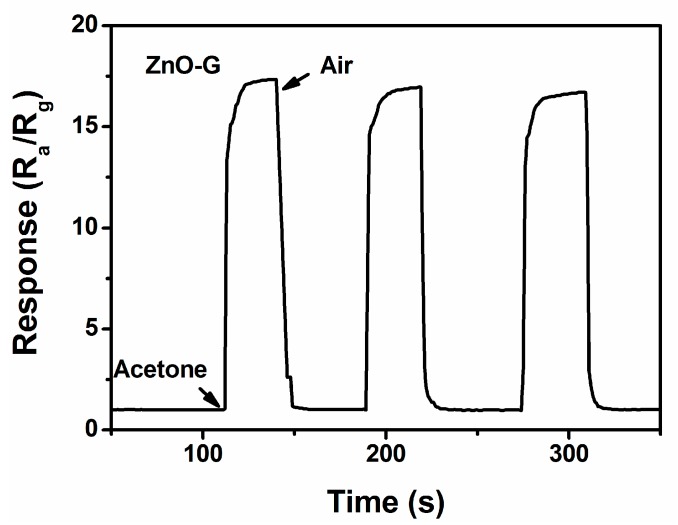
The reproducibility of the ZnO-G sensor on successive exposure (three cycles) to 100 ppm acetone at 280 °C.

Further tests for the reproducibility of the sensor based on ZnO-G is illustrated in Figure 10. It is revealed that the sensor maintains its initial response amplitude without a clear decrease upon three successive sensing tests to 100 ppm of acetone, albeit the swift response and recovery process, indicating that the sensor has an outstanding stability throughout the cycle test.

## 4. Discussion

**Figure 11:**
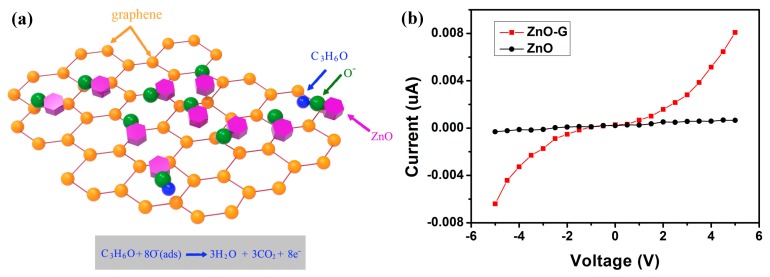
(**a**) The scheme of the proposed gas sensing mechanism: the adsorption behavior of acetone molecules on the ZnO-G nanocomposite; (**b**) Representative I–V curves for ZnO-G and ZnO sample.

According to literature [32,33], the most probable explanation for such behavior could be as follows. The response of metal-oxide semiconductor sensors is mainly determined by the interaction of a target vapor and the surface of the metal-oxide material. The following reactions may occur in the surface reaction (Figure 11a) [34]. When acetone vapor (CH_3_COCH_3_) reacts with oxygen species (O^−^) on the surface of a metal-oxide material, it is oxidized to carbon dioxide and water. This liberates free electrons in the ZnO conduction band, leading to a decrease in the resistance of an n-type semiconductor. Two reasons may account for the better performance exhibited by the ZnO-G compared to pure ZnO sensors. Firstly, due to the high specific surface area (2600 m^2^/g [35]) of graphene, mixing graphene could increase the amount of surface active sites for the adsorption of target gas, which contribute to the improvement of sensitivity and detection of acetone in a wide range. Secondly, the introduction of ZnO-G could improve the electron-transfer rate due to the high electron mobility of graphene and the increase in the surface area of the sensing materials due to the two-dimensional structure. With the high charge carrier mobility, graphene provides direct conduction paths for carriers to transport from the junction to the external electrode. I–V curves indicate that the incorporation of graphene films into the ZnO particles significantly optimizes the material’s conductivity (Figure 11b). For the ZnO-G sample, a relatively larger current (on the order of µA) passes through the film. The calculated resistivity of the ZnO sample is higher than that of the ZnO-G sample. Therefore, the electrical signals link closely and propagate rapidly, which accelerate the response and recovery process of the ZnO-G based sensor.

## 5. Conclusions

This paper describes a simple liquid phase separation method for the fabrication of exfoliated graphene and a wet chemical synthesis route for ZnO-G composites. The effects of graphene doping on the acetone sensing properties of ZnO-G have been investigated. Evaluation of gas-sensing properties revealed that the sensor based on ZnO-G exhibits more outstanding selectivity, higher response, and faster recovery behaviour toward acetone in contrast to those based on pure ZnO. A response of 13.3 to 100 ppm acetone is obtained at 280 °C. Our results indicate that graphene can significantly improve the acetone sensing properties of ZnO, which has excellent potential applications in gas sensors.
